# Mediastinal bronchial artery aneurysm presenting as an incidental mediastinal mass: A rare finding

**DOI:** 10.1016/j.radcr.2021.06.076

**Published:** 2021-08-01

**Authors:** Nizar EL Bouardi, Baderddine Alami, Ghada El Mounceffe, Meriam Haloua, Youssef Alaoui Lamrani, Meryem Boubbou, Mustapha Maaroufi

**Affiliations:** a Radiology department, Hassan II university hospital, Fez, Morocco; b Faculty of medicine and pharmacy, Sidi Mohamed Ben Abdellah University, Fez, Morocco

**Keywords:** Mediastinal bronchial artery, Aneurysm, Transtarterial embolization

## Abstract

Mediastinal bronchial artery aneurysm is very rare and only few cases have been reported in the literature. The clinical presentations are varied, ranging from an incidental radiological finding to a cataclysmic rupture leading to hemorrhagic shock. Thus, a quick treatment is indicated upon diagnosis. Therapeutic options are various including surgical resection, stent grafting with percutaneous embolization of feeding vessel or transtarterial embolization. Herein we describe a case of an incidental mediastinal bronchial artery aneurysm in a 63-year-old man, managed by transtarterial embolization.

## Introduction

Bronchial artery aneurysms are rare. Their localization is either pure pulmonary, mediastinal or double. Their clinical presentations are mainly linked to their size and location, and largely depends on the underlying etiology. Their rupture is a frequent mode of revelation, and it engages the patient's vital prognosis. Classical therapeutic methods include thoracotomy surgery and embolization. We will trough this manuscript reports a case of a giant mediastinal left bronchial artery aneurysm treated by transtarterial embolization and we will also compare this technique to the different therapeutic methods reported in the literature.

## Case presentation

**Fig. 1:**
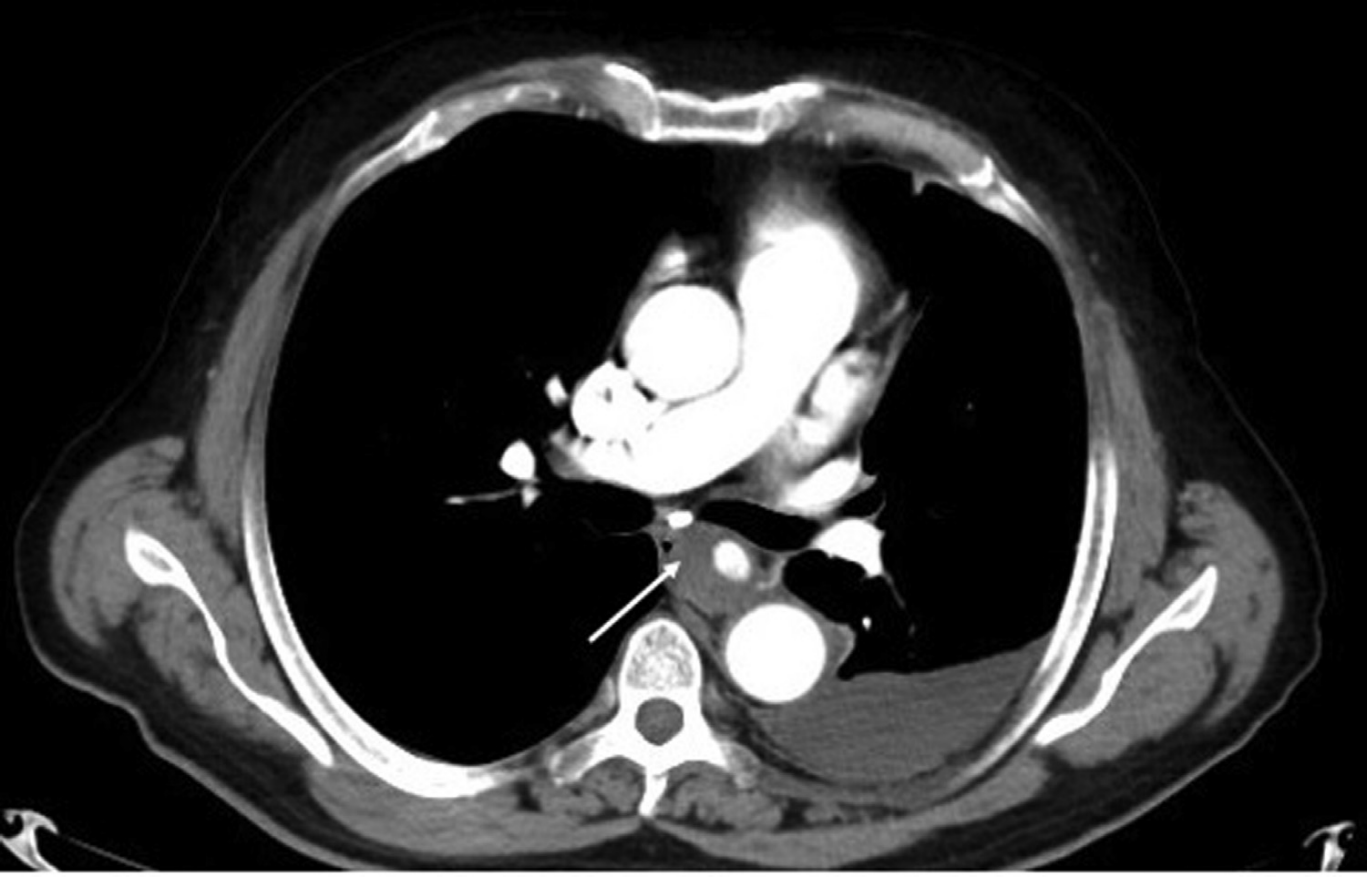
Axial CT scan view in the arterial phase showing a mediastinal mass enhancing the same as aorta (arrow). Notice the moderate pleural effusion (Liquid density 10 HU).

**Fig. 2:**
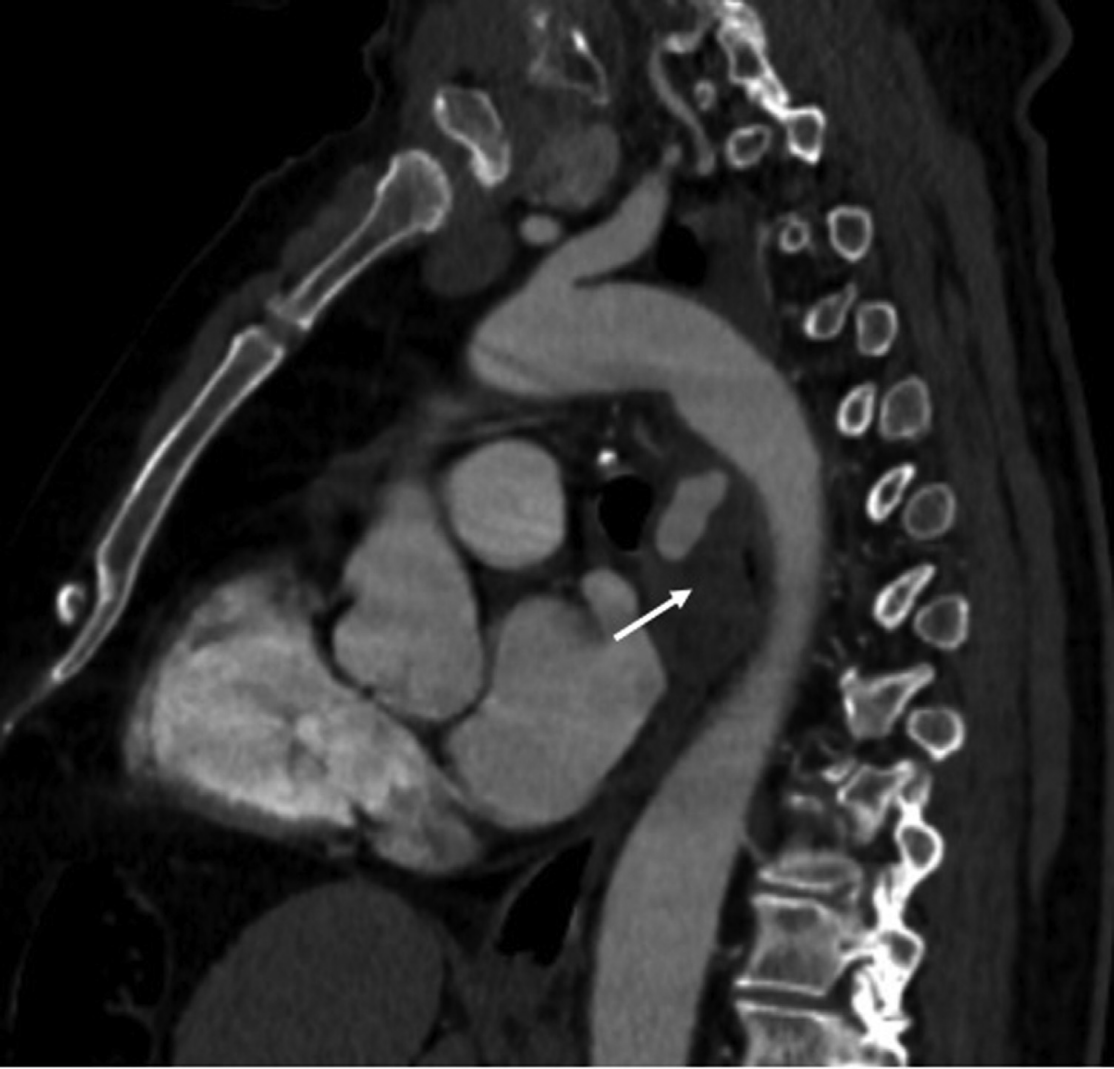
sagittal CT scan view in the arterial phase showing a mediastinal partially thrombosed aneurysm (arrow). There was no evidence of communication with the descending aorta excluding the hypothesis of an aortic aneurysm.

**Fig. 3:**
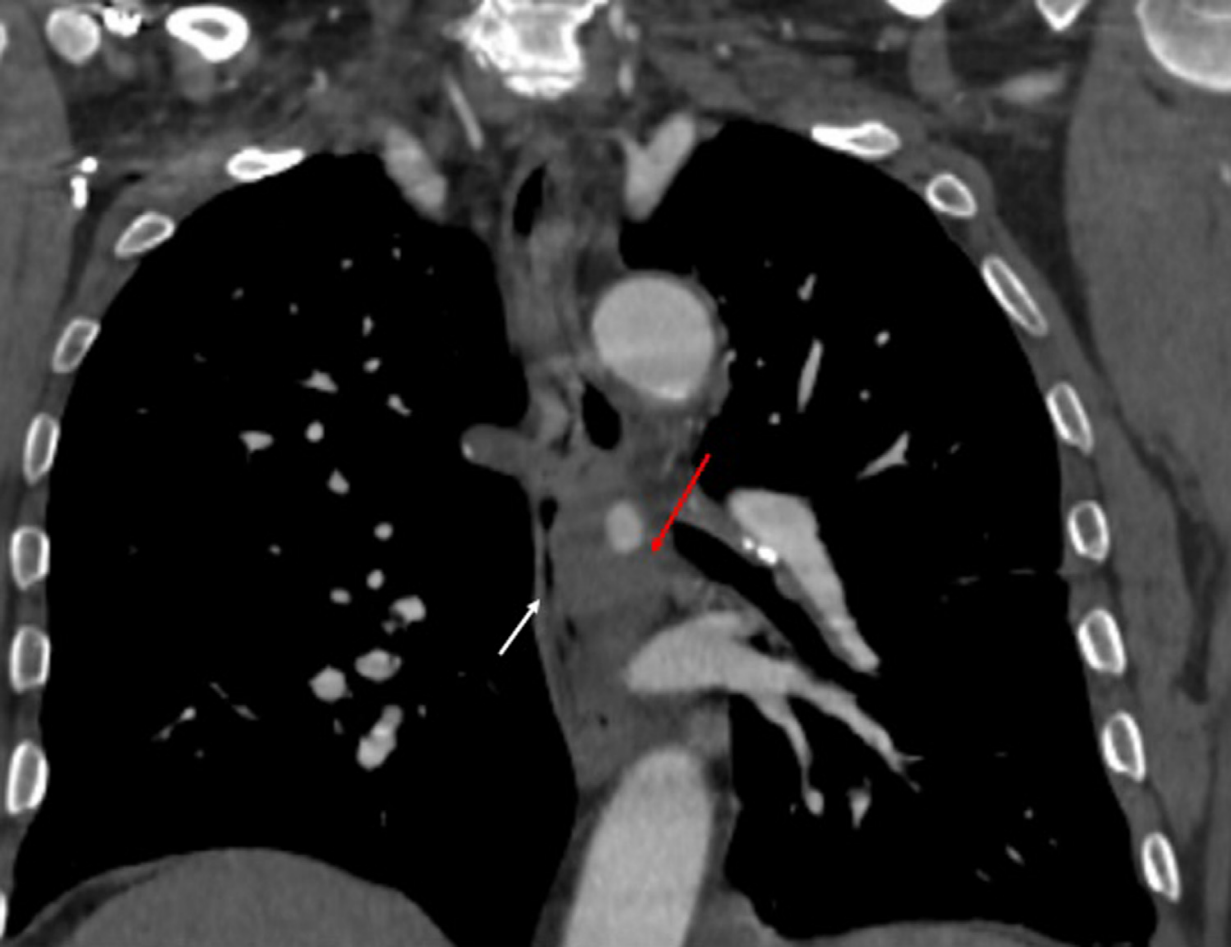
Coronal CT scan view showing the relation of the aneurysm with mediastinal structures. It is displacing the esophagus (red arrow). to the right.

**Fig. 4:**
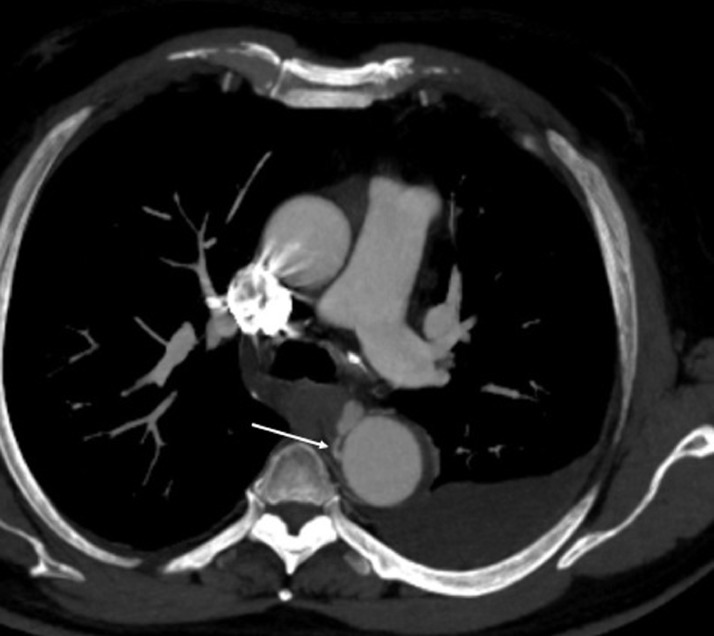
Axial CT scan view in the arterial phase showing the left bronchial artery feeding the aneurysm (arrow).

A 63 years old man, operated 10 years ago for an inguinal hernia, underwent a chest CT scan in the context of a chronic cough. There were no parenchymal abnormalities except a moderate left pleural effusion (liquid density 10 Hounsfield unity). We incidentally found a round 25 mm diameter mediastinal mass adjacent to the descending aorta. Its upper pole was at the carina. (Fig. 1, Fig. 2). It displaced the esophagus to the right (Fig. 3). The lesion was enhanced with contrast agent to the same degree as the aorta, which revealed that this lesion was vascular the findings were mimicking a saccular aneurysm of the descending thoracic aorta. However, this lesion seemed to be separate from the descending aorta, without any communication. Instead, small tubular structure emanating from the descending aorta was identified in the space between the descending aorta and feeding the mass (Fig. 4). This was interpreted as being the left bronchial artery. On the basis of these findings, a definitive diagnosis of mediastinal bronchial artery aneurysm was made.

**Fig. 5:**
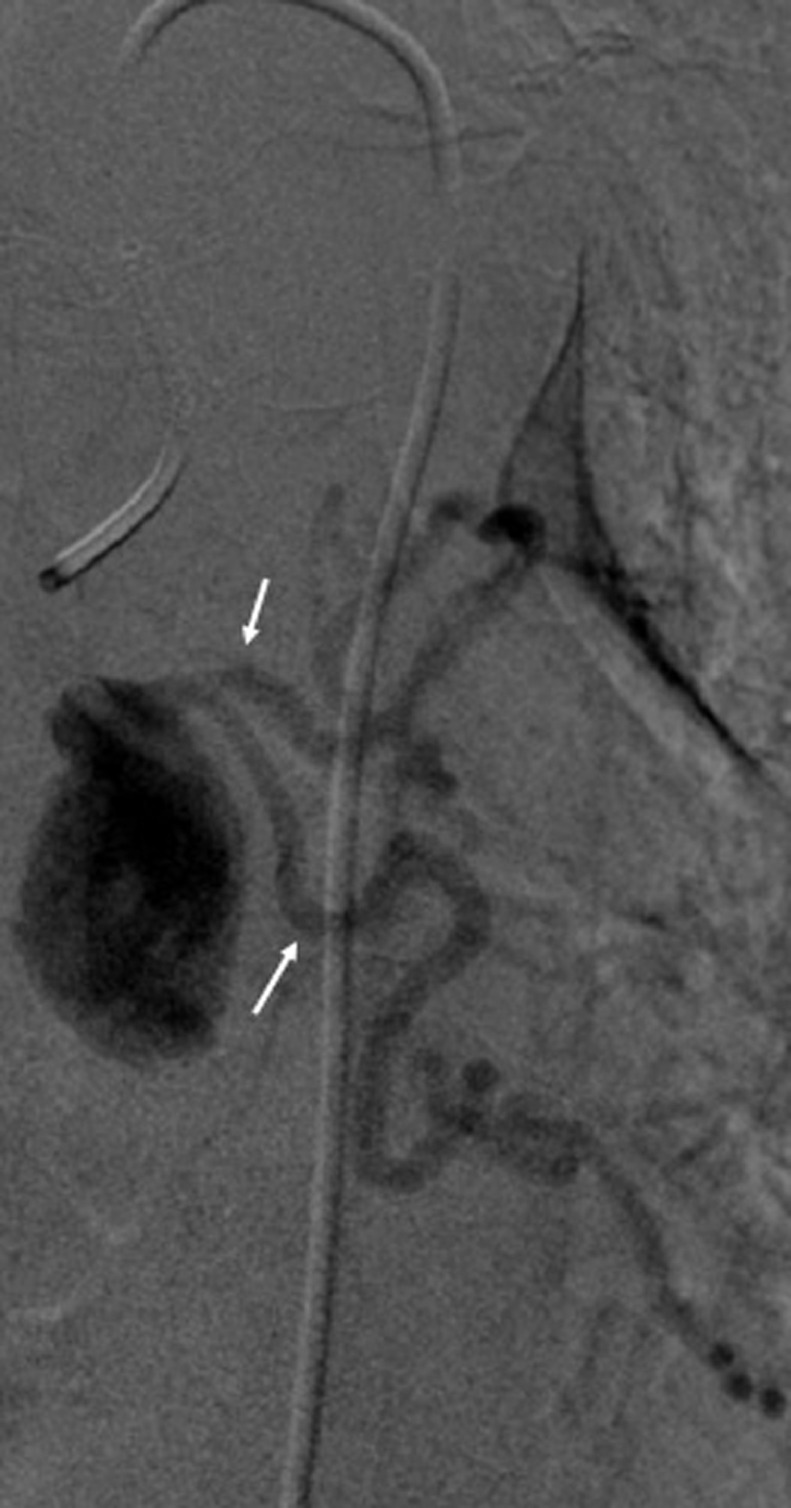
angiography view showing the catheterization of the bronchial artery ostium. The aneurysm was distant 15 mm from the ostium and extended to the hilar branches (arrow).

**Fig. 6:**
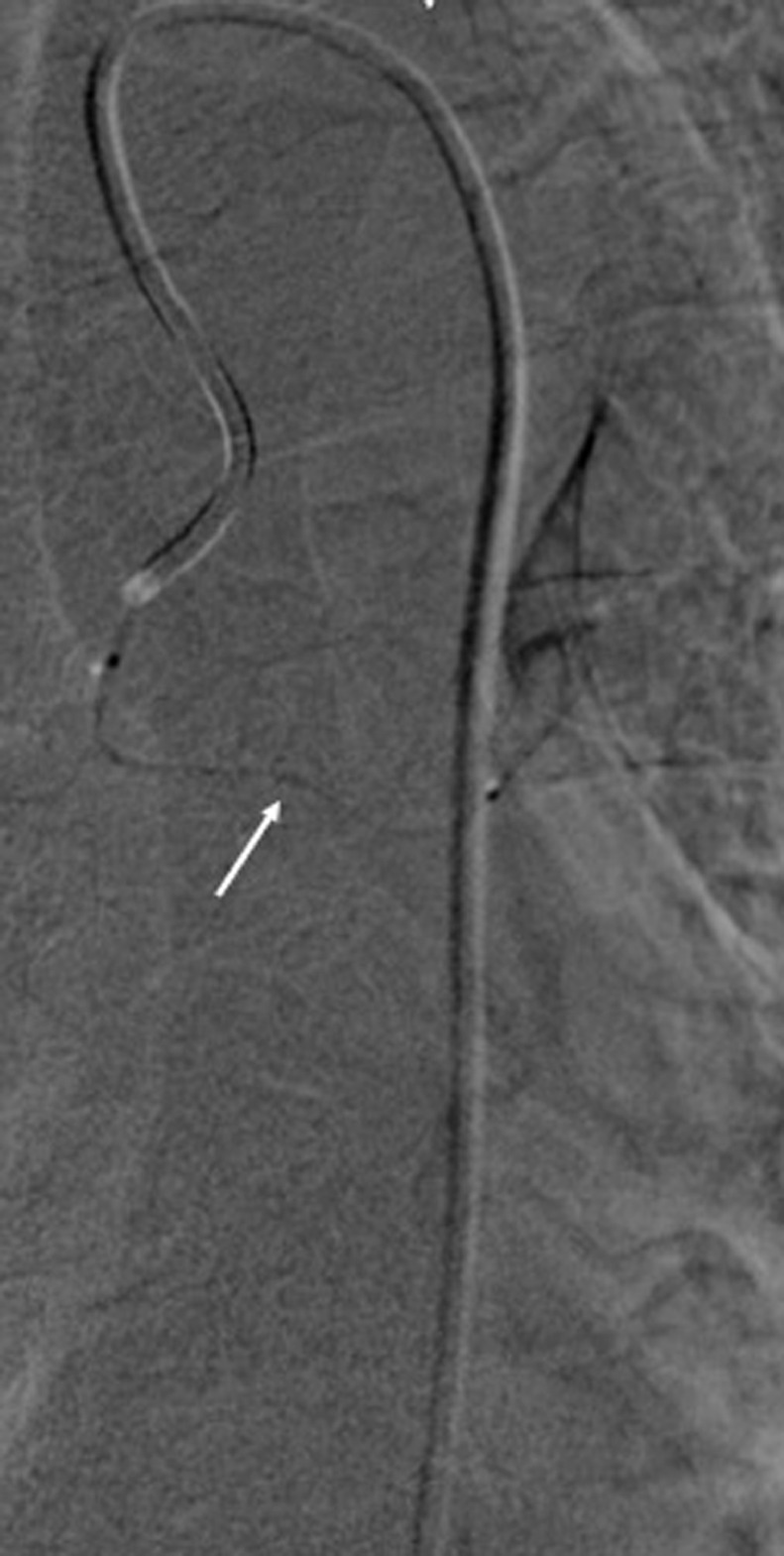
Angiography showing Ultra selective catheterization of the bronchial artery with a 1.4 French microcatheter (arrow).

**Fig. 7:**
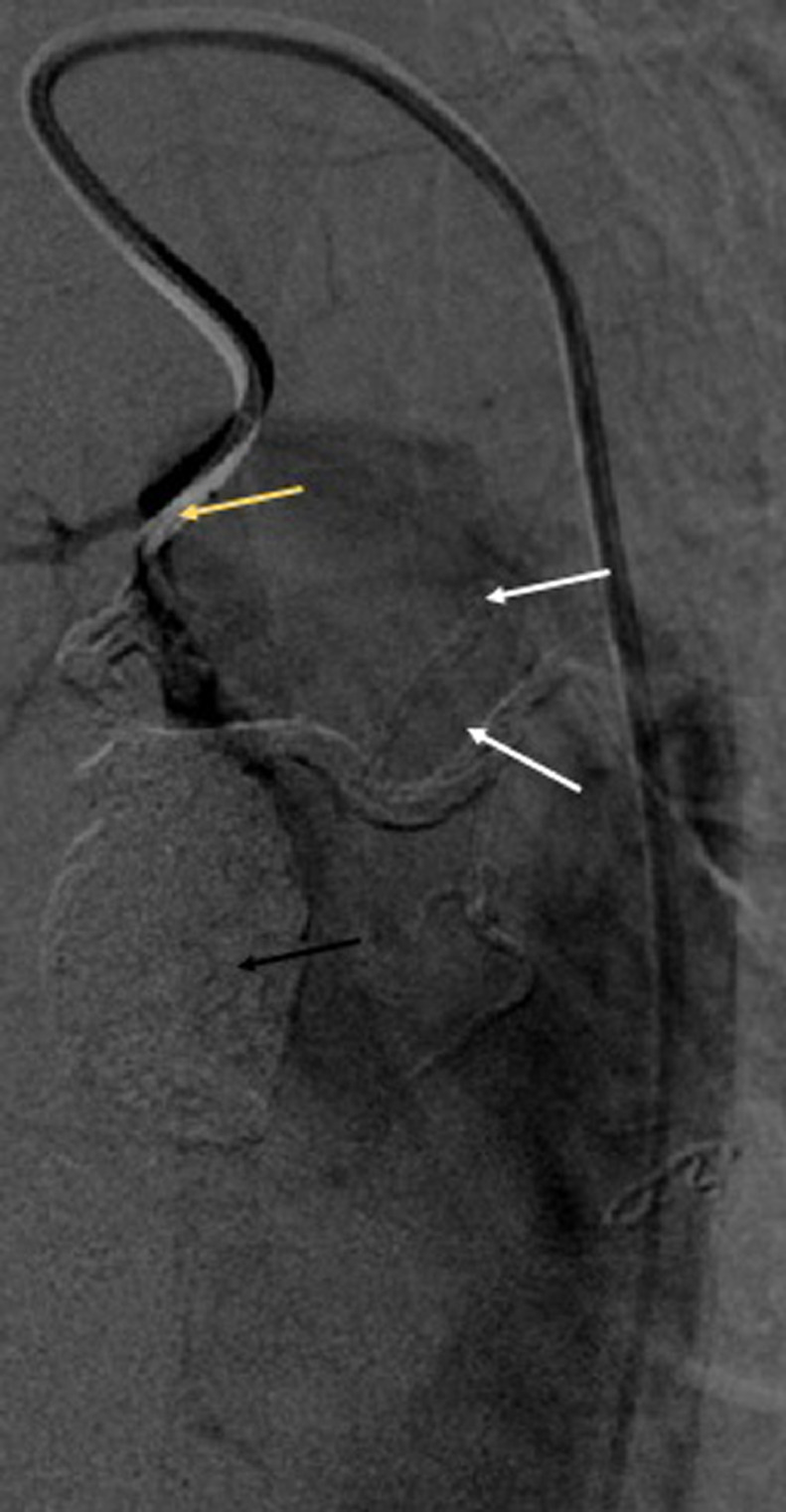
Angiography showing final control after embolization of the aneurysmal sac (black arrow), the hilar branches (white arrows) and the bronchial artery (yellow arrow).

This aneurysmal sac was partially thrombosed. It was 15 mm distant from the ostium of the left bronchial artery and extended to the hilar branches (Fig. 5). There was no sign of rupture or fissuration. The patient was asymptomatic with no history of pulmonary infection or trauma or CT evidence of atherosclerosis. There was also no history of vasculitis or radiotherapy. After a multidisciplinary consultation including interventional radiologist, thoracic surgeon and pulmonologist, a collegial decision to treat this aneurysm with a transtarterial embolization was taken. After a selective catheterization using a 1,4 French microcatheter (Fig. 6), we embolized using Onyx* the aneurysmal sac and also the hilar branches and the left bronchial artery proximal to the aorta to ensure the occlusion of the inflow and the outflow and prevent the recurrence Immediate control was satisfying showing a total exclusion of the aneurysm (Fig. 7).

## Discussion

Bronchial artery aneurysms are divided into 2 entities: mediastinal and intra pulmonary aneurysms. Mediastinal aneurysms are very rare and uncommon, with a reported incidence less than 1% based on selective bronchial angiograms [1].

Their physiopathology remains uncertain. The cases reported in the literature mention congenital cases occurring in a context of sequestration [2] and pulmonary agenesis [3], atherosclerotic aneurysms of the thoracic aorta [4] or secondary to inflammatory lung diseases and bronchiectasis [5,6] or further to trauma [7]. Finally, more rarely, certain cases are associated vascular abnormalities such as Rendu Weber-Osler disease [8] and septic conditions such as mediastino pulmonary tuberculosis [9]. In approximately one quarter of described cases, no predisposing etiology could be identified. In our patient, there were no signs of an inflammatory disease since the inflammatory assessment was normal. The possibility of a post-traumatic or iatrogenic aneurysm was not selected either, as he never had a catheterization of the aorta or any previous notion of thoracic trauma. We think that it was an idiopathic aneurysm.

Mediastinal bronchial artery aneurysms are generally silent. They are often discovered incidentally by chest imaging as in our case. Most of symptomatic aneurysm are giant. They can be revealed either by a mediastinal compression syndrome, mainly in the form of a superior vena cava syndrome [10], dysphagia by esophageal compression, or by an acute rupture simulating the severe pain of aortic dissection or an aortic rupture.

This rupture can be intrathoracic (hemothorax), intra mediastinal (hemomediastinum) or in a neighboring organ (esophagus, veina cava, bronchi). This complication is frequently inaugural and it is a life-threatening condition.

Multiphasic computed tomography is the gold standard for the diagnostic. It is done in the supine position, including non-enhanced, arterial phase and venous phase acquisitions after intravenous injection of iodine contrast agent. It allows an exhaustive study of the aneurysm thus making it possible to measure its neck, its diameter, its distance from the ostium and the hilar branches as well as the complications: lumen thrombosis, fissuration and rupture

Once diagnosed, these mediastinal bronchial artery aneurysms should be immediately treated. Their rupture is unpredictable and unrelated to the aneurysm diameter [11].

Surgery for mediastinal artery aneurysms bronchial consists of resection of the aneurysm. It's a risked surgery that is often performed under extracorporeal circulation requiring thoracotomy and aortic clampage. Postoperative complications are frequent, associating the postoperative risks specific to thoracotomy as well as the risks associated with aortic clamping on fragile ground, as was the case with our patient. The length of hospital stay and the cost are high, surgery is then reserved for low-risk patients and / or with contraindications to endovascular treatment.

Endovascular treatment is now increasingly the most recommended treatment. It has the same efficiency as the surgical treatment with less morbidity and mortality. The choice depends on the morphological features of the aneurysmal sac. If the bronchial artery proximal to the aneurysm has sufficient length, transtarterial embolization using coils, biological glue (N-Butyl cyanoacrylate) or onyx is the method of choice. The success of endovascular therapy depends on the ability to isolate the aneurysm. Both the outflow and inflow to the aneurysm need to be occluded, as retrograde flow through the outflow vessel may result in recurrence. In this case, a second embolization or surgical resection should be done. However, it may sometimes be difficult to safely perform transtarterial embolization of bronchial artery aneurysms that arise directly from aorta with no or short neck. This anatomic challenge may be overcome by the use of detachable coils, which are useful for precise placement without prolapse into the aorta and are retrievable if the coil is not properly deployed [12]. Recently, thoracic stent-grafting in conjunction with percutaneous embolization has been used to treat mediastinal bronchial artery aneurysms not anatomically suitable for embolization alone [13].

## Conclusion

Mediastinal bronchial artery aneurysms can be successfully treated with currently available endovascular methods. The immediate success rate, the low rate of complications postoperative and the short duration of hospitalization in comparison with conventional surgical techniques and the long-term effectiveness are in favor of endovascular treatment.

## Patient consent

Consent was obtained from the patient. The study was conducted anonymously.

## Availability of data and materials

The data sets are generated on the data system of the university hospital of Fès.
